# P02.151. Effect of Ayurvedic treatment in diabetic sensory polyneuropathy: a non-randomized, observational clinical study

**DOI:** 10.1186/1472-6882-12-S1-P207

**Published:** 2012-06-12

**Authors:** P Kalapi, M Patel, C Mehta, C Kessler, S Gupta

**Affiliations:** 1 J.S.Ayurved College, Nadiad, Gujarat, India; 2 Charité Medical University, Berlin, Germany

## Purpose

Diabetic sensory neuropathy is a common complication affecting approximately 30% of patients with diabetes mellitus. Conventional drugs are used for symptomatic relief only and, moreover, have certain side effects. In Ayurveda, various treatment modalities for diabetic sensory polyneuropathy have been described in detail and are being used successfully in routine Ayurvedic care.

## Methods

Thirty-three patients with clinical features of diabetic sensory polyneuropathy confirmed both clinically and with a neuropathy analyzer machine were included. All patients received Ayurvedic treatment as outpatients or inpatients (depending on the severity) for one month. Treatment included oral administration of Phyllanthus niruri powder 3g twice a day and Abutilon indicum root decoction 40 ml twice a day. Patients also received Ayurvedic dietary advice (in particular avoidance of spicy, sour, deep fried, hot and refrigerated food items). Patients were assessed for changes in clinical features based on subjective scoring at admission and at the time of completion of the treatment. The neuropathy analyzer machine was used for recording sensory perception of vibration and cold and heat sensations in the feet. Data were analyzed statistically by using the student’s t-test.

## Results

All 33 patients completed Ayurvedic treatment. The results showed significant (p<0.001) relief in numbness (70.2±0.7%), tingling sensations (72±0.5%), burning sensations (77.6±0.8%) and pain in lower limbs (64±0.5%). There were also significant (p<0.001) improvements in right and left foot sensory perception of vibration (31.2±8.7% and 32.6±8.7%, respectively), cold sensation (19.7±5.1% and 23.1±6.4%) and heat sensation (6.9±3.9% and 5.2±3.9%).

## Conclusion

The results of this pilot study point towards a possibly effective and safe Ayurvedic treatment approach for diabetic sensory neuropathy. Larger trials are warranted to support these findings.

